# Hemi-Versus Total Hip Arthroplasty in Femoral Neck Fractures? Predicting Failure on a 10-Year Data Analysis of the German Arthroplasty Registry (EPRD)

**DOI:** 10.3390/jcm14051457

**Published:** 2025-02-21

**Authors:** Sven Hungerer, Florian Hinterwimmer, Iris Leister, Severin Langer, Alexander Gotzler, Claudio Glowalla

**Affiliations:** 1 BG Trauma Center Murnau, 82418 Murnau, Germanyclaudio.glowalla@bgu-murnau.de (C.G.); 2 Institute for Biomechanics, Paracelsus Medical University Salzburg, 5020 Salzburg, Austria; 3 Department of Orthopedics and Sports Orthopedics, School of Medicine and Health, TUM University Hospital, Technical University of Munich, 80333 Munich, Germany; florian.hinterwimmer@tum.de; 4 Institute for AI and Informatics in Medicine, School of Medicine and Health, TUM University Hospital, Technical University of Munich, 80333 Munich, Germany; 5 Spinal Cord Injury Center, BG Trauma Center Murnau, 82418 Murnau, Germany; iris.leister@bgu-murnau.de

**Keywords:** femoral neck fracture, hip fracture, arthroplasty registry, EPRD, machine learning, hemiarthroplasty, total hip arthroplasty, cemented fixation, feature importance

## Abstract

**Background/Objectives:** The German Arthroplasty Registry (EPRD) recorded almost 100,000 femoral neck fractures between 2013 and 2023. The aim of this study was to identify survival rates and risk factors for failure in individuals with femoral neck fractures. **Methods:** A dataset of 97,410 cases from the EPRD was analyzed. We compared hemiarthroplasty (HA) and total hip arthroplasty (THA) using machine learning algorithms (MLAs) and statistical modeling approaches. For the MLA, the dataset was partitioned into training and test sets, with iterative feature selection and hyperparameter search. Predictive models were developed using XGBoost classifiers. Based on the feature importance, we performed LASSO regression to assess the odds ratios for key predictors of implant failure. **Results:** The failure rate was 3.7% for HAs and 5.6% for THAs, with a peak six weeks after surgery. LASSO regression revealed six risk factors for failure: non-cemented stem fixation (OR: 1.022, 95% CI: 1.019–1.026), treatment type (THA vs. HA; OR: 1.013, 95% CI: 1.010–1.016), time to discharge (OR: 1.006, 95% CI: 1.006–1.006), male sex (OR: 1.003, 95% CI: 1.000–1.005), age (OR: 0.999, 95% CI: 0.999–0.999), and day of surgery (weekday vs. weekend/holiday; OR: 1.004, 95% CI: 1.002–1.008). **Conclusions:** Longer hospital stays, male sex, and surgeries performed on weekends or holidays were associated with higher failure risks, while cemented fixation and hemiarthroplasty showed protective effects. Given that the overall failure rate was only 4.5%, even a 1–2% change in odds represents a very large clinical effect.

## 1. Introduction

The guidelines and regulation from the social security statute book in Germany recommend the surgical treatment of patients with femoral neck fractures within 24 h [1]. In 2021, the vast majority of patients in Germany with fractures near the hip joint were older than 70 years (>87%), resulting in arthroplasty being the predominant treatment option for these fractures in contrast to an osteosynthesis [2]. The aim of surgery in this mostly geriatric and vulnerable patient population is to enable immediate postoperative mobilization and return to unrestricted activity. Therefore, the choice of treatment techniques remains the subject of ongoing debate, particularly concerning the type of prosthesis and the method of anchoring, which vary depending on the age group. Key considerations include whether to perform hemiarthroplasty (HA) or total hip arthroplasty (THA), as well as the decision between cemented and cementless fixation [3,4]. For example, the bone cement implantation syndrome is one risk factor for increased perioperative mortality in individuals who underwent surgery with cemented techniques [5], while the risk for periprosthetic fractures is higher in those with uncemented stem fixation [6]. These discussions are influenced by factors such as patient age, bone quality, comorbidities, and anticipated functional outcomes, underscoring the complexity of tailoring the optimal surgical approach for each individual.

Machine learning’s ability to handle complex and large-scale datasets, such as those from clinical registries, has made it an invaluable tool in orthopedic research ranging from radiographic analysis to outcome analysis [7] or advancing personalized treatment strategies in geriatric care [8]. In a previous study by Gotzler et al., a dataset from 2013 to 2020 was analyzed using a multivariate approach on half the amount of data [9]. The aim of the present study was to enhance the prediction accuracy of outcomes in this study population by applying machine learning and LASSO regression analysis in an expanded dataset, nearly doubled in size and extending to 2023. The primary target parameters were the treatment options (HA or THA) and the choice between cemented vs. cementless stem fixation on the revision rates and survival outcomes following femoral neck fractures. Furthermore, the analysis included identifying risk factors and assessing their impact on failure rates and the subsequent need for revision surgery, as well as overall mortality.

## 2. Materials and Methods

The German Arthroplasty Registry (EPRD), an initiative of the German Society for Orthopaedics and Orthopaedic Surgery (DGOOC), is a nationwide multi-center registry that prospectively collects data from participating hospitals in Germany.

**Figure 1:**
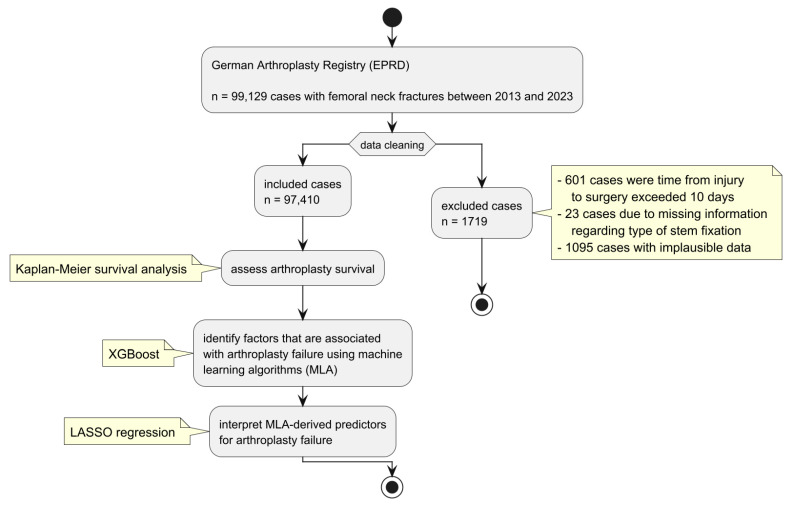
Study flowchart with data processing and analysis.

The EPRD has provided the registry data for hip arthroplasty after femoral neck fractures (ICD 10 D2022 S72.0* [10]). From the initial dataset of 99,129 cases in the EPRD-registry (spanning 2013 to 2023), we applied several exclusion criteria to ensure data validity and relevance (Figure 1). First, 601 cases were excluded due to the time from injury to surgery exceeding 10 days. Next, 23 cases were removed due to missing information regarding fixation type (cemented vs. non-cemented). Additionally, we excluded 1095 cases with implausible data entries, including discrepancies in vital status, reasons for transfer or discharge, inconsistencies between observation time and durability, and instances where arthroplasty was labeled as “intact” despite durability being less than the observation time. After applying these criteria, the final sample size for this study was 97,410 cases.

The dataset included patient-specific data (age at surgery, sex, and Elixhauser Comorbidity Score (ECS)); procedure-specific data (i.e., date of surgery and number of cases of the treating hospital); and implant-specific data type of arthroplasty (THA or HA), stem fixation technique, and failure. The time in days is the “durability” period, during which the primary treatment could be observed before a revision procedure, i.e., failure occurred [11].

Further parameters were added during the 10-year period, such as ECS or treatment numbers of the participating hospitals, so that the absolute number may change with the parameter under consideration. The ECS is calculated from a patient’s existing ICD diagnoses. The index is suitable for estimating mortality and the resources likely to be required during a hospital stay. The higher the score, the more severely ill the patient [12]. Nationwide public holidays and weekends days were retrospectively identified and matched with the date of surgery in order to assign patients to two groups: weekday vs. weekend/holiday.

All statistical analyses and figures were completed in R (R Core Team, 2023, version 4.2.3; run in Windows 10 × 64) or SPSS (Version 26, IBM). Descriptive statistics for continuous variables and frequency counts for categorical variables were calculated. To test the association between categorical and continuous variables, χ^2^ or Student’s *t*-test was applied as appropriate. The level of significance was set at α = 0.05.

### 2.1. Survival Analysis

To assess arthroplasty survival and compare failure rates over time, we performed a Kaplan–Meier survival analysis with log-rank tests to evaluate differences between subgroups, including treatment type (THA vs. HA), stem fixation method (cemented vs. uncemented), and surgical timing (weekday vs. weekend/holiday). Patients were censored at the time of data extraction from the registry based on social insurance records if no-failure event had occurred by then. Kaplan–Meier curves were generated to visualize time-to-failure distributions. Statistical analyses were conducted using appropriate survival analysis functions within R and SPSS.

### 2.2. Machine Learning Algorithms (MLAs)

For the MLA, the data were partitioned into training and test sets to enable clean model evaluation and ensure the validity of the results. The partitioning was stratified to maintain the balance of positive and negative samples, preventing bias in model performance due to class imbalance. Labels were defined based on implant durability and failure status. A case was classified as a “failure” if the implant failed within 730 days (2 years) of observation. Conversely, cases where the implant did not fail within 730 days were labeled as “no-failure”. The time of 730 days was chosen because more than 95% of failures occur in the first two years after surgery.

A total of 265,000 feature combinations were initially considered, leading to a refined selection of the best-performing combination. The final model was trained using the XGBoost classifier [4], with hyperparameters tuned through a grid search. The hyperparameter grid included the number of estimators (50, 100, or 150); maximum tree depth (3, 5, or 7); learning rates (0.01, 0.05, 0.1, or 0.2); and test size ratios (10%, 15%, 20%, or 25%). Additionally, the scale_pos_weight parameter was adjusted to account for class imbalance, with values calculated as the ratio of negative to positive samples in the dataset.

The explainability of the model was assessed using feature importance scores generated by the XGBoost classifier. These scores quantified the contribution of each feature to the reduction in loss during training. The analysis identified the most influential features for the prediction. The model’s performance was evaluated using two key metrics: accuracy and the area under the receiver operating characteristic curve (AUC). Accuracy measured the overall correctness of the model’s predictions, while AUC evaluated the model’s ability to discriminate between failure and no-failure cases across varying thresholds. The best model was selected based on the highest AUC score [5].

### 2.3. Regression Analysis

Based on the feature importance produced by XGBoost, we performed LASSO (least absolute shrinkage and selection operator) regression for the endpoint failure, including the following parameters: time to hospital discharge (=duration of hospitalization), day of the week when surgery was performed (weekday vs. weekend/holiday), cases per year in the treating hospital, type of treatment (total hip arthroplasty [THA] vs. hemiarthroplasty [HA]), type of stem fixation (cemented vs. not cemented), sex, and age. The number of cases per year in the treating hospital was not assessed in approximately one-third of cases and was heavily penalized by the LASSO regression. Therefore, this variable was excluded from the final model.

To ensure robust validation and interpretability of the MLA-derived predictors, we applied LASSO regression, which facilitates feature selection while minimizing overfitting. This approach helps in identifying the most relevant predictors while maintaining model stability. The resulting odds ratios (ORs) and confidence intervals (CIs) provide clinically interpretable associations between selected predictors and the endpoint. To further enhance statistical robustness, we employed a bootstrapping approach using the bootLasso function from the HDCI package in R, performing residual-based bootstrapping with 500 resamples (B = 500). Confidence intervals were computed at the 95% level (alpha = 0.05). Model selection was performed via 10-fold cross-validation to determine the optimal regularization parameter for LASSO regression. The model included an intercept, standardized the predictors for comparability, and did not penalize the intercept.

## 3. Results

### 3.1. Descriptive and Univariate Statistics

**Figure 2:**
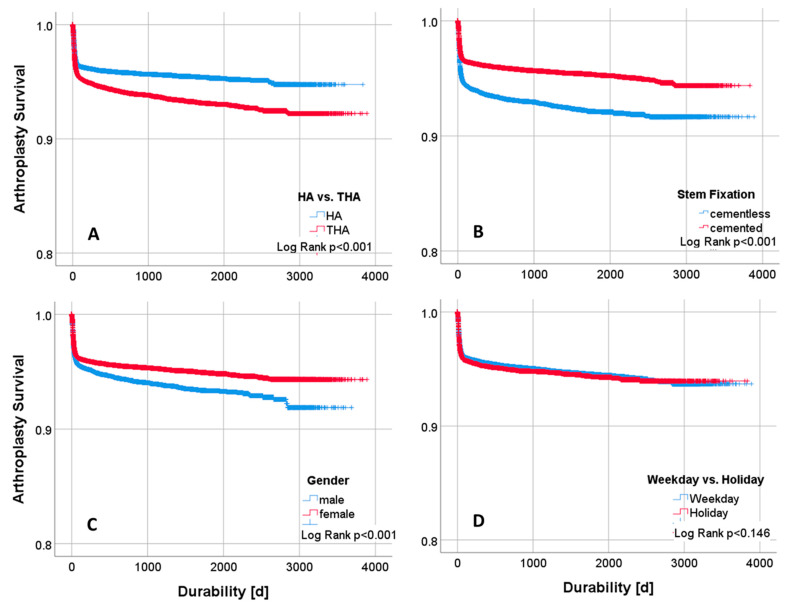
Kaplan–Meier plots with log-rank testing on arthroplasty survival and time to revision surgery (durability). (**A**) HA vs. THA, (**B**) cementless vs. cemented stem fixation, and (**C**) male vs. female patients were significant (log-rank, *p* < 0.001). (**D**) Surgery on weekdays vs. holidays/weekends showed no significant difference in survival analysis (log-rank, *p* = 0.146).

**Figure 3:**
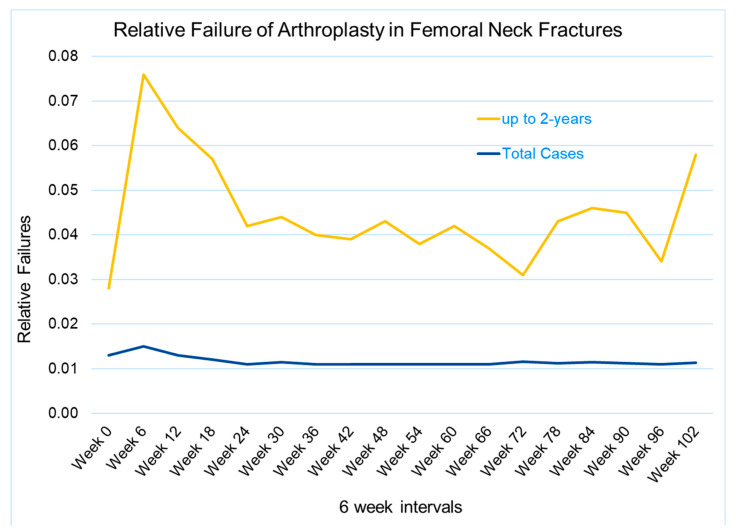
Relative failure distribution of the arthroplasties with focus on the first two years of observation (yellow line) and with focus on all cases (blue line). Failure occurs with a peak in the first 6 weeks.

A total of 97,410 cases were analyzed, 71% of which were women and 29% men (Table 1). The average age was 81 ± 9.4 years. In the registry, 4428 (4.5%) failures were documented. The failure rate for men was significant, with 5.1% (*n* = 1441) vs. 4.3% for women (*n* = 2987) (Figure 2C, *p* < 0.001). The relative revision rate peaked in the first 6 weeks after surgery (Figure 3).

The German guidelines require a surgical treatment of hip fractures within 24 h [1]. In the registry, the time between injury and surgery was 0.99 ± 1.11 days. The average time to discharge was 13.2 ± 7.9 days and was significantly higher in patients with failure (23.0 ± 17.7 days (*p* < 0.001)).

HA was implanted in 67% of cases and THA in 33%. The failure rate for THA was 6.0% vs. 3.9% for HA (Figure 2A, *p* < 0.001). A cementless stem fixation technique was used in 23% of the cases and a cemented technique in 77% (Table 1). The failure rates for cementless fixation were significant higher at 6.8% vs. cemented stem at 3.9% (Figure 2B, *p* < 0.001).

In the current registry data, the median durability, i.e., time from surgery to revision, was only 24 days (0–2843 d) and, without revision, 833 days (0–3888 d), i.e., the median observation time recorded in the insurance system for individuals without failure. A durability of zero days indicates immediate implant failure on the day of surgery or the patient died at day of surgery (Table 1).

Overall, 23% of arthroplasty procedures were performed on a weekend or public holiday (Table 1). The performance of operations on weekends or public holidays showed no significant difference compared to operations on weekdays, with a focus on failure rate (*p* = 0.253) in pairwise unadjusted comparisons and in log-rank testing (Figure 2D).

The number of cases in the treating hospitals might also have an effect on the risk of failure. Clinics with less than 99 arthroplasty procedures per year have a failure rate of 3.9% and clinics with 100 to 200 procedures have a rate of 3.7% (Table 1). Interestingly, clinics who did not share their data on expertise had a higher failure rate of 5.9%.

The mean ECS was 7.3 ± 7.6 points and increased significantly (*p* < 0.001) to 8.1 ± 8.0 in patients with arthroplasty failure (Table 1).

### 3.2. Machine Learning Algorithm

The final model was trained using a set of features that included “time to discharge”, “weekday”, “holiday and weekend”, “type of treatment”, “sex”, “age decade”, “number of cases per year”, and “type of stem fixation”. This feature set, combined with the optimized hyperparameters, yielded the best predictive performance. The model achieved an accuracy of 0.7664 and an AUC of 0.7463. The best hyperparameters included a learning rate of 0.01, a maximum tree depth of 5, 150 estimators, and a scale_pos_weight of 22.1435. The dataset was split into 78,817 training cases and 19,705 testing cases.

**Figure 4:**
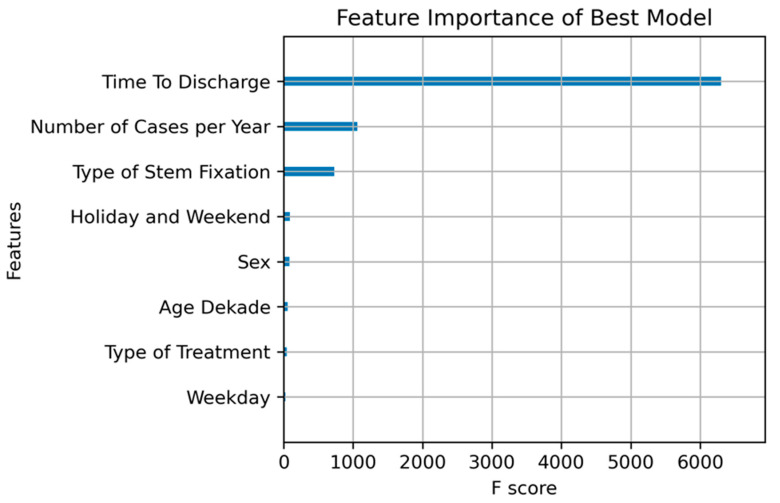
MLA showed the feature importance of time to discharge as the most influential feature in predicting implant failure, followed by holiday and weekend, type of treatment, and number of cases per year. These features significantly contributed to the model’s ability to distinguish between failure and no-failure cases.

Feature importance analysis indicated that “time to discharge” was the most influential feature in predicting implant failure, followed by “holiday and weekend”, “type of treatment”, and “number of cases per year” (Figure 4). These features significantly contributed to the model’s ability to distinguish between failure and no-failure cases. Other features, such as “type of stem fixation” and “weekday”, also contributed to the model’s predictive power, though their relative importance was lower compared to the top predictors. The findings underscore the relevance of discharge timing, treatment type, and patient demographics in understanding implant durability.

### 3.3. Predictors for Failure

**Figure 5:**
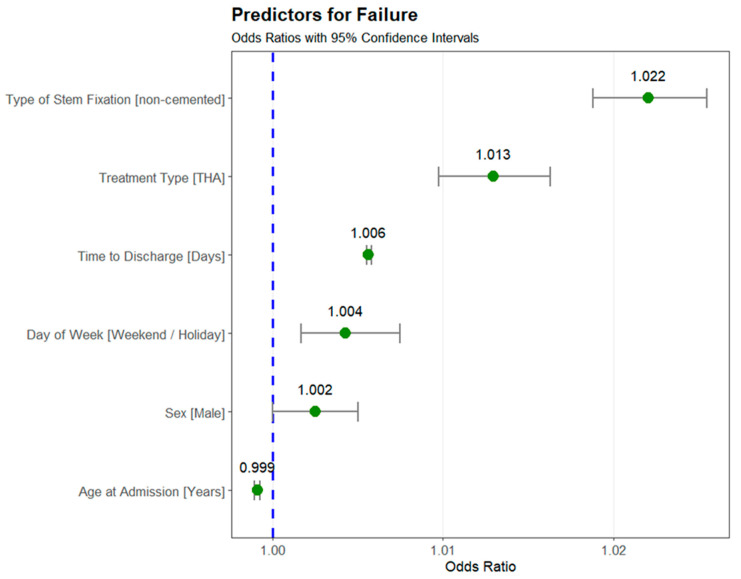
Predictors for failure of arthroplasty in femoral neck fractures are given as odds ratios with 95% confidence intervals. The highest risk for failure is a cementless stem fixation, followed by the treatment with THA compared to HA. Other predictors for failure were time to discharge, surgery on a weekend or holiday, and male sex (blue dotted line is an odd ratio of 1.0, green dots on the right are increased risk of failure, left reduced risk for failure). All included predictors are statistically significant.

The final LASSO regression model, informed by feature importance scores from the XGBoost classifier, identified six predictors for the endpoint failure. The odds ratios (ORs) and their 95% confidence intervals (CIs) for the predictors are presented in Figure 5.

Non-cemented stem fixation was associated with higher odds of failure, with an odds ratio of 1.022 (95% CI: 1.019–1.026) compared to cemented stem fixation. Type of treatment (THA vs. hemiarthroplasty) was also associated with a higher odd of failure in patients treated with THA, with an odds ratio of 1.013 (95% CI: 1.010–1.016) compared to those undergoing hemiarthroplasty.

Time to discharge was significantly associated with failure, with an odds ratio of 1.006 (95% CI: 1.006–1.006), indicating that longer hospital stays are related to an increased risk of failure. Similarly, male sex was associated with a small but statistically significant increased risk of failure (OR: 1.002, 95% CI: 1.000–1.005).

Age at admission was associated with a slightly reduced odds ratio of 0.999 (95% CI: 0.999–0.999), indicating a minimal protective effect with increasing age.

Interestingly, the day of week when the surgery was performed (weekday vs. weekend/holiday) showed an odds ratio of 1.004 (95% CI: 1.002–1.008) and, therefore, an increased risk of subsequent failure for surgeries performed on weekends or holidays compared to those performed on weekdays.

All predictors in the final model were statistically significant, as indicated by confidence intervals that did not cross 1. The results highlight that non-cemented stem fixation, THA, and time to discharge were among the strongest predictors of failure, while age and surgery performed on a weekday showed protective associations.

## 4. Discussion

The present study is a cumulative evaluation of the German Arthroplasty Registry (EPRD) with regards to risk factors for prosthesis failure in patients with femoral neck fractures and arthroplasty treatment in the last decade. The present study uses MLA followed by LASSO regression analysis to identify complex dependencies of risk factors on the survival rate of hip implants after treatment of femoral neck fractures.

### 4.1. Time of Surgery or Number of Cases

The “weekend effect” on the outcome of hip fracture patients is controversial. Most of the studies on “weekend effect” on hip fracture outcomes focus on hospital mortality, complication rates [13,14], or surgical delay [15]. The current data showed a higher odds ratio for failure of the arthroplasty if surgery is performed on a weekend or a holiday, although the groups did not differ significantly in the common statistical analysis. This finding is supported by the MLA in a previous analysis from the German Arthroplasty Register [9]. In this analysis, the MLA already showed an increased feature importance of the time of surgery but was not significant in multivariate regression analysis. The present modeling revealed the correlation of weekday surgery with a better outcome. The guidelines and regulation from the social security statute book in Germany recommend the earliest possible treatment of patients with femoral neck fractures within 6 to 24 h for arthroplasty [1]. This implies that treatment during the weekend or public holidays is common, although weekday surgeries might have better outcomes for the patients.

In the present registry study, the number of arthroplasty surgeries per year performed by the hospital had no significant impact on the outcome of hip fractures. In contrast, the EPRD data showed a decrease in the probability of failure with higher hospital case numbers [16]. This was supported by data from the British Registry in THA, which found that high-volume surgeons have lesser revision rates [17], but the data are still not consistent; the Norwegian registry showed no influence on the survival rate of the arthroplasty in correlation with the experience of the surgeon [14]. Additionally, the value of the data might be another obstacle. One-third of the clinics did not share their numbers of arthroplasty surgery per year. In these hospitals, the failure rate was higher than the other two-third who reported their data. Therefore, a complete dataset might reveal correlations.

### 4.2. Failure Sex and Age

Sex-specific bone morphologic differences are supposed to be the reason for the higher incidence of femoral neck fractures in elderly female patients [18,19]. In contrast to the pathogenesis of femoral neck fractures is the finding that, after surgery, male sex and younger age are risk factors for failure [9]. These findings are confirmed by our data. One reason for a higher failure rate might be an increased risk of infection for men [11]. Data from the Dutch arthroplasty register showed similar findings, that men and age below 80 years are risk factors for prosthesis failure [20]. An MLA approach on the Nordic Arthroplasty Register Association dataset showed similar findings, with a higher risk for periprosthetic fracture in men with uncemented stems [21]. Another reason might be the selection bias of the EPRD data registry. The first-line treatment for patients under 60 years of age is osteosynthesis within 6 h to 24 h, depending on the nationwide guidelines. Therefore, in the EPRD, there are only younger patients included with more comorbidities or other risk factors, leading to a treatment of the hip fracture with an arthroplasty.

### 4.3. Cemented or Cementless Technique

The Cochrane Database of Systematic Reviews state a benefit for cemented HA. Cemented stems have a lower risk for periprosthetic fractures but a higher risk for pulmonary embolism [22]. The initial ability for weight bearing might be one reason for a better postoperative recovery in cemented techniques [23]. The American Joint Replacement Registry (AJRR) recommends that restoration with a HA should be performed with a cemented stem, and a THA can be done cementless [24]. Nevertheless, cementless stem fixation has higher revision rates due to periprosthetic fractures [20,22,25,26,27]. The EPRD recommends the cemented technique for patients aged above 75 years [11]. A recent study on German healthcare data combined with EPRD data showed that there was no difference in 5-year mortality between cemented and cementless treatment. For the cemented fixation, a 1% higher in-hospital mortality was found, maybe attributable to a bone cement implantation syndrome [5]. A similar finding is the significantly higher mortality of patients with cemented HA in the first 48 h [28]. Nevertheless, the advantages for the cemented stem fixation outweigh the cementless technique. The elderly patients are less likely to suffer periprosthetic fractures or dislocation with cemented arthroplasty, and quality of life is better in these patients [22,29]. The lower rates of major complications for cemented HA might be one reason for better cost-effectiveness of this technique [30].

### 4.4. THA or HA?

In our clinical practice, the algorithm for femoral neck fractures determines the use of cemented HA for patients above 80 years and cementless THA for patients under 70 years of age, leaving a gray area between 70 and 80 years of age. Decisions were made according to the “biological age” of the patient. Women were more likely chosen for cemented technique because of the prevalence of osteoporosis. Most registry data support cemented HA for the treatment of elderly patients [22], and only one Swedish registry study showed a lower revision rate for THA compared to HA [31]. The annual reports of the EPRD and the data of the Italian National Registry showed lower revision rates for HAs compared to THAs in femoral neck fractures [32,33]. The reasons for revision surgery in the cementless technique are predominantly infection and periprosthetic fractures [34]. Gotzler et al. found, in a previous analysis of EPRD data, an increasing risk for younger men (<60 y) with a high Elixhauser Score for cementless arthroplasty [9]. The current analysis confirms the findings that younger age, male sex, and a cementless THA are predictors for failure and raise the question of whether the algorithm for femoral neck needs to be adapted.

### 4.5. Time of Failure

Data from the New Zealand registry showed two peaks for revision after arthroplasty. Early revision in the first year and after more than 10 years [35]. The data of the Swedish Arthroplasty Registry indicate that the probability of a reoperation in the three first years after primary surgery is the largest in the first year after elective THA [36]. The first two years after surgery are also a quality indicator in the Swedish Registry. Again, the reasons for early revision surgery are infection, periprosthetic fractures, and dislocation. The differentiation in these categories of failure is not possible in our dataset.

### 4.6. Statistics

Univariate analysis using Kaplan–Meier curves and chi-square test is common practice in statistical analysis [37]. The multivariate analysis showed that all final models performed significantly better than the respective null model, which was confirmed by chi^2^ and F statistics. This means that, generally, relevant factors could be identified, most of which have a significant influence on the target variables. The prognostic contribution of the multivariate models and the recorded predictors is rather low. This suggests that the actual, data-generating process is either not captured by the models or is only revealed to a limited extent. Another important point is that the numerous interactions, the strength of which are difficult to estimate, make both the statistical analyses and the adjustment of the models considerably more difficult.

The selected ML model (XGBoost) can use FI to weigh a specific variable with regards to the occurrence of an event—in this case, the probability of failure [38]. In this study, the service life of the prosthesis, BMI, and age had the highest FI for the probability of failure. Increased BMI and long service life have already been described as risk factors for arthroplasty in several studies [39,40,41].

MLAs, and especially XGBoost, are increasingly the subject of studies on the prediction of various events. Initial studies are aimed at creating risk profiles for patients and better estimating possible pre-, intra-, or postoperative complications [42,43].

### 4.7. Limitations

Although the dataset included 97,410 cases, the target parameter failures occurred in “only” 4428 cases. This leads to a so-called class imbalance, which is a typical characteristic for medical data. Any additional variable reduces the number of observed failures in the groups, reducing the discrimination of the groups and increasing the multiplicity, i.e., the fact that carrying out several comparisons and statistical tests makes a random correlation appear causal [44]. Another “limitation” is the competing risk of death, i.e., the older the patients are, the more likely they are to die before the revision, and thus, the survival time of the arthroplasty is reached [45]. Therefore, the MLA was trained on the dataset with an observation time of two years to minimize the bias of death, but this might lead to another imbalance of the data analysis.

Some of the parameters were added after the initial start of the EPRD in 2013. For example, height and weight beginning in 2017 or the number of reported hospital cases. Therefore, these data are not consistently available for all cases [33]. The number of reported hospital cases was not available in approximately one-third of the cases. Therefore, the algorithm must be capable of dealing with missing data points. Nevertheless, it cannot be ruled out that the final result was distorted by missing data. XGBoost can also be considered a black box, which means that interpreting and understanding the predictions can be difficult [46]. A future aspect is the growing amount of data in the following years, allowing the algorithm to be trained and become more accurate in the analysis of the feature importance of these data.

## 5. Conclusions

The current registry analysis of the German Arthroplasty Registry (EPRD) on the treatment of femoral neck fractures with HA or THA shows that the highest impact on failure of the arthroplasty is a non-cemented stem, followed by THA in contrast to HA. After surgery, relative failure of the arthroplasty occurs with a peak after 6 weeks. This might be one reason why an increase in “time to discharge” is predictive of failure. This present dataset allows us to reveal a correlation for failure if surgery takes place on a weekend or holiday. Male sex is a risk factor for failure.

The increasing number of cases in national registries provides an ideal field for the use of MLAs to analyze these correlations in large datasets such as the EPRD. To enhance clinical applicability, MLAs can be integrated into preoperative planning by enabling patient-specific risk stratification based on factors like implant type, fixation method, and demographics. This allows for personalized risk assessment and informed decision-making. Additionally, MLA predictions can be incorporated into decision support systems, providing real-time risk estimates to assist surgeons in selecting the optimal treatment strategy. These applications could improve surgical outcomes and contribute to data-driven, precision medicine approaches in orthopedic surgery.

## Figures and Tables

**Table 1:** Patient-specific data with age on admission, sex, Elixhauser Score, etc. are given in means ± standard deviation and median with range. Treatment-specific data such as treatment type (HA vs. THA) and stem fixation (cemented vs. uncemented) are presented as numbers and percentages.

	Intact	Failure	Overall	*p*-Value
	(*n* = 92,982)	(*n* = 4428)	(*n* = 97,410)	
Age at Admission (Years)				
Mean (SD)	81.0 (9.31)	78.9 (9.95)	80.9 (9.35)	<0.001
Median [Min, Max]	82.0 [24.0, 111]	81.0 [33.0, 103]	82.0 [24.0, 111]	
Sex				
female	66,111 (71.1%)	2987 (67.5%)	69,098 (70.9%)	<0.001
male	26,871 (28.9%)	1441 (32.5%)	28,312 (29.1%)	
Time between Injury and Surgery (Days)				
Mean (SD)	0.988 (1.11)	0.999 (1.17)	0.988 (1.11)	0.545
Median [Min, Max]	1.00 [0, 10.0]	1.00 [0, 10.0]	1.00 [0, 10.0]	
Time to Discharge (Days)				
Mean (SD)	13.2 (7.92)	23.0 (17.7)	13.6 (8.85)	<0.001
Median [Min, Max]	11.0 [0, 136]	18.0 [0, 152]	11.0 [0, 152]	
Treatment Type				
Hemiarthroplasty (HA)	63,055 (67.8%)	2525 (57.0%)	65,580 (67.3%)	<0.001
Total Hip Arthroplasty (THA)	29,927 (32.2%)	1903 (43.0%)	31,830 (32.7%)	
Type of Stem Fixation				
non-cemented	21,274 (22.9%)	1547 (34.9%)	22,821 (23.4%)	<0.001
cemented	71,708 (77.1%)	2881 (65.1%)	74,589 (76.6%)	
Durability (Days)				
Mean (SD)	833 (751)	124 (316)	833 (751)	<0.001
Median [Min, Max]	632 [0, 3888]	24 [0, 2843]	590 [0, 3890]	
Day of Week—Surgery				
weekend/holiday	21,225 (22.8%)	1044 (23.6%)	22,269 (22.9%)	0.253
weekday	71,757 (77.2%)	3384 (76.4%)	75,141 (77.1%)	
Number of Cases per Year				
1–99	38,947 (41.9%)	1597 (36.1%)	40,544 (41.6%)	<0.001
100–200	23,486 (25.3%)	913 (20.6%)	24,399 (25.0%)	
more than 200	89 (0.1%)	1 (0.0%)	90 (0.1%)	
not assessed	30,460 (32.8%)	1917 (43.3%)	32,377 (33.2%)	
Elixhauser Comorbidity Score				
Mean (SD)	7.2 (7.5)	8.1 (8.0)	7.3 (7.6)	<0.001
Median [Min, Max]	5.0 [−14.0, 56.0]	6.0 [−11.0, 44.0]	5.0 [−14.0, 56.0]	
Geriatric Complex Treatment				
no	71,632 (77.0%)	3474 (78.5%)	75,106 (77.1%)	0.030
yes	21,350 (23.0%)	954 (21.5%)	22,304 (22.9%)	
Vital Status				
alive	49,810 (53.6%)	2235 (50.5%)	52,045 (53.4%)	<0.001
deceased	43,172 (46.4%)	2193 (49.5%)	45,365 (46.6%)	

## Data Availability

Registry data are accessible at the German Arthroplasty Registry (EPRD—https://www.eprd.de) accessed on 28 June 2023.

## Abbreviations

The following abbreviations are used in this manuscript:

ECS	Elixhauser Comorbidity Score
EPRD	German Arthroplasty Registry
FI	Feature Importance
FNF	Femoral Neck Fracture
HA	Hemiarthroplasty
MLA	Machine Learning Algorithms
THA	Total Hip Arthroplasty
